# CRISPR-Cas9-mediated exon skipping as a cardioprotective strategy in Duchenne muscular dystrophy

**DOI:** 10.1016/j.omtm.2023.08.013

**Published:** 2023-09-04

**Authors:** Harry Wilton-Clark, Toshifumi Yokota

**Affiliations:** 1 Department of Medical Genetics, University of Alberta, Edmonton, AB, Canada

Duchenne muscular dystrophy (DMD) is a debilitating and fatal genetic disease characterized by systemic muscle breakdown affecting both skeletal and cardiac muscle.^1^ Typically, skeletal muscles are affected first in early childhood, leading to a loss of ambulatory ability by the early teens. As the disease progresses, the cardiac muscles become increasingly affected leading to DMD-related cardiomyopathy and eventual death, with most patients living until their mid to late twenties. Given its well-characterized genetic pathogenesis, numerous precision therapies have been explored as potential treatments for DMD, and both exon skipping therapy and microdystrophin therapy have been granted FDA approval in recent years.^2^^,^^3^ While these therapies have shown promise in their ability to treat the skeletal phenotype of DMD, they (particularly exon skipping therapy) have shown minimal cardiac benefit. Thus, there exists an urgent unmet medical need for therapeutics that can treat or prevent DMD-related cardiomyopathy.

In this issue, Rok et al. describe their work focused on treating DMD-related cardiomyopathy using AAV-mediated CRISPR-Cas9.^2^ Working in a mouse model containing a deletion of *DMD* exons 52–54, the authors used CRISPR-Cas9 to induce the excision of *DMD* exon 55, leading to the production of a truncated but in-frame protein product. Treatment of neonatal Δ52-54 mice with their optimized AAV9-delivered CRISPR construct led to significantly improved cardiac dystrophin expression and amelioration of the cardiac phenotype compared to controls.

Primarily, the work of Rok et al. serves as an exciting proof-of-concept study for a CRISPR-based DMD therapeutic with demonstrated cardiac efficacy.^2^ As previously mentioned, effective cardiac therapies for DMD are sorely needed by the clinical community. In response, numerous approaches have been undertaken by the research community including stem cell-based therapies, utrophin modulators, and peptide-conjugated oligonucleotide therapies, all of which display marked cardiac benefit compared to existing therapies.^4^^,^^5^^,^^6^ Notably among these options, CAP-1002 (a cardiosphere-derived cell therapy) and rAAVrh74.MCK.GALGT2 (a utrophin modulator) are in phase III and phase I/II clinical trials, respectively, and CAP-1002 has already demonstrated cardiac benefit in DMD patients in its phase II findings.^7^^,^^8^ The results from Rok et al. provide preliminary evidence to suggest that CRISPR-based therapies may also serve as viable cardiac therapies for DMD, and future studies will be required to better assess their efficacy, as well as to compare their benefit to the other cardiac therapies in development.

Secondarily, Rok et al. help to further characterize the Δ52-54 mouse model. A limitation in most DMD mouse models is that they fail to demonstrate the cardiac abnormalities expected in human patients, limiting researchers’ ability to develop effective cardiac therapies.^9^^,^^10^ Δ52-54 mice, a new model developed previously by their group, display a cardiac phenotype resemblant of hypertrophic cardiomyopathy (HCM).^2^^,^^10^ While less common in DMD patients than dilated cardiomyopathy, the measurable HCM in Δ52-54 mice suggests that this could be a valuable model for all murine DMD researchers. By demonstrating that dystrophin restoration can ameliorate the observed phenotype, Rok et al. have validated their model for use in therapy development, offering improved infrastructure for their and others’ cardiac-focused DMD research.
